# Diagnosis of the Internal Secretory Disorders

**Published:** 1916-05

**Authors:** Henry R. Harrower

**Affiliations:** M. D., F. R. S. M. (Lond.), Los Angeles, California


### The Diagnosis of the Internal Secretory Disorders.

BY HENRY R. HARROWER, M. D., F. R. S. M. (LOND.), LOS ANGELES, CALIFORNIA.

### I.

### PRELIMINARY CONSIDERATIONS—THE FREQUENCY OF INTERNAL SECRETORY DISORDERS IN GENERAL PRACTICE.

The insidiousness and practical importance of disturbed function of the glands of internal secretion is far greater than most physicians realize; and the frequence with which it may be encountered in almost every phase of general as well as special practice, coupled with the fact that many times it is entirely overlooked, constitute the excuse for this series of articles.

Increasing attention is being paid to the study of the glands of internal secretion, or *endocrinous organs*, as we shall call them, and rightly so. Our knowledge of the physiological action of these organs has been acquired almost entirely in the last fifty years; and has been augmented very materially in the past fifteen or twenty years. In fact, practically all we know of the internal secretions has been learned in this brief period and the establishment of the fundamental contentions of the now famous Claude Bernard and Brown-Sequard was not accomplished and their experiences scientifically explained until as recently as 1902, when Starling of London suggested the term "hormone" (from the Greek word which means "I arouse," or "I set in motion") to designate a class of chemical substances, of which his newly discovered secretin was the type, which are produced in various parts of the body and carried by the blood or other body fluids to various remote organs, where they excite certain physiological manifestations, thus correlating the functions of numerous and widely separated organs.

The subject is of much more than academic importance for "treatment based on the internal secretions is, in some instances, positively startling in its results, and bids fair to revolutionize our methods in several lines of practice; it is also eminently satisfying from a scientific viewpoint, being far removed from our old hesitating empiricism."*

The endocrinous glands, then, are factors of no mean import in the maintenance of that balance of activities which we usually

*Editorial in the *New York Medical Journal*, February 26, 1916, p. 412.

call "health," and hence are worthy of more careful study and practical consideration in our clinical work. These organs and their hormones play a much more vital part than many physicians have allowed themselves to think. Variations in their activities deserve the closest attention, for too often endocrinous disorder is only thought of when there is obvious disease of one or more of these glands. Since they are now definitely known to control growth and development, regulate metabolism and dominate the nervous system, more especially the sympathetic or autonomous system, their widespread activities assume a greater importance, for it is quite clear that these hormones are altogether indispensable to the maintenance of the physiological harmony of the body.

Too often we are prone to look upon this class of disorders as rare and occasional and their diagnosis as comparatively easy because of the obviousness of such well marked diseases as cretinism, myxedema, giantism or acromegaly. This is a mistake, for functional disorders of this class are of every day occurrence and are naturally far more important than the more obvious organic diseases, for they are still in their earliest stages before serious disharmony has been caused; and, of course, are more responsive to suitable treatment.

Such all-essential factors in the regulation of human chemistry should be of interest to every general practitioner in his investigation of *every* condition in which disordered function is present. We should not be satisfied to know how to diagnose and treat those definite cases of definite endocrinous disease, but rather should we be always on the lookout to detect and understand the importance of the insignificant and minor aberrations from the normal, for in so doing in many a case we may be able to forestall the more serious and more obvious diseases which, if left alone, may later assert themselves.

As the larger functions of the internal secretions are being appreciated, their influence both for good and for bad is seen to extend far beyond the expected limits of definite endocrinous disease, for as one writer has aptly put it: "There are a number and variety of conditions which can be understood and properly treated only after full comprehension of the work of the endocrinous glands." All the uncounted clinical and experimental experiences which have been directed as the solution of the numerous problems which this ever-broadening subject has opened up, have convincingly demonstrated that the influence of the various units of the endocrinous system, as well as of the system as a whole, is far more extensive and complex than even the best posted physiologists had supposed, and that many phenomena credited to nervous or sympathetic nervous influences were really the result of hormonic disharmony. In fact, we know as a result of the painstaking work of Cannon, Crile, Elliot, and Sergent, that as the sympathetic system is under the direct control of one or more of these hormone influences, disorders with prominent sympathetic disturbances, as shock, collapse, hysteria and other neuroses, may be traced further back than we have been in the habit of doing heretofore, and, what is of far greater practical importance, may be controlled by applying the principles which the study of this subject simultaneously has proved possible in the domain of therapeutics. Crile's exhaustive study of the kinetic system—the adrenals, thyroid, brain and muscles—and its practical application in what he chooses to call "anoci-association," is but one of many profitable phases of this huge subject, a part of which it is proposed to consider in this series.

Now that the interest of the aggressive section of the profession is being focussed upon these endocrinous glands, and much regarding their study and the importance of the many phases of the subject is appearing in current medical literature, a comparatively new branch of medicine is gradually being differentiated, and with the better knowledge that we have of the physiology of the hormone-producing organs, there comes not merely an increased diagnostic skill, but a broadened therapeutic horizon, for in the study of the internal secretions lies the future of the treatment of most functional disorders. To tell the truth the extensive ramifications of this subject and the increasing prominence of the endocrinous features in so many minor as well as important disorders, is awakening an interest in a new specialty which Sajous has recently called *"hemadenology"* (blood-gland-discourse) and which the writer prefers to designate *"endocrinology"* (internal-secretion-discourse). As Sajous says, this branch of medicine "claims the right to exist as a specialty for its field is greater in scope than some which have earned well merited recognition."* Sajous then continues: "Its influence on the improvement of the race through the light it will shed upon the pathology of the unfit, mental and physical, cannot but prove a blessing. If to this we add the many disorders it will serve to elucidate through

*"C. E. de M. Sajous, "Hemadenology: "A New Specialty," *New York Medical Journal,* February 20, 1915, p. 365.

collective effort on the part of the host of investigators it is bound to enlist * * * the day may come when the inauguration of hemadenology may be considered as having marked a new epoch in medicine."

All who have studied this subject admit that it has a fascination that cannot be measured. The profitable applications that have been made in clinical practice by the employment of organotherapy, or "hormone therapy," explain in a good measure the favor with which this subject is being received by the medical world.

As we occupy ourselves in searching for the earliest signs of endocrinous disorders automatically we gain a better insight into the intricacies of the functions of the body and are not merely able to forestall the later and more serious organic disease, but so often we run across associated manifestations of the most diversified kinds, from nocturnal enuresis to chilblains or from neurasthenia to a stiff neck, which may be modified directly by suitable organotherapeutic measures which we may be directing at an associated but entirely different condition.

None can deny that a knowledge of these twin subjects—endocrinology and hormone therapy—has put an entirely new aspect on the outcome of many intractable disorders. The increasing appreciation of the role of the endocrinous glands has, as Leonard Williams has said, "lightened our darknesses and shown us miracles." What other word than "miracle" can be applied to the startling effects of thyroid feeding upon the pitiful conditions of cretinism? How many of the "darknesses" of practical medicine are being illuminated by what science has taught us regarding the hormones in physiology and therapeutics, may be more evident as this series unfolds.

In the words of an editorial writer in *American Medicine*:* "Many a chronic and intractable disorder is due to an overlooked defect in the production of the hormones of the internal secretory glands. Increasingly greater stress is being laid upon the importance of these chemical messengers, and there is now little doubt that in health as well as in disease they regulate and correlate the metabolic activities of the body." Again the importance of this is emphasized in a recent review in the *Lancet*† from which we quote: "As our knowledge has progressed, the influence of the ductless glandular system has proved to be far wider and more

*Editorial in *American Medicine*, August, 1915, p. 590.
†Book review in the *Lancet*, December 11, 1915, p. 1301.

penetrating than any of the earlier investigators suspected. It controls growth and metabolism, and, in short, determines largely the nature of that factor which the older physicians spoke of as 'constitution.'"

The internal secretions have revolutionized physiology and with it clinical medicine, just as hormone therapy has revolutionized certain phases of treatment; hence the general practitioner, above all others, stands to benefit by this added fund of knowledge. As we become better acquainted with the endocrinous disorders it will be noticed that intimations of their presence are staring at us in every turn in our daily routine; and we will find many an occasion to congratulate ourselves that we have taken time to investigate this fascinating and interesting subject.

715 Baker-Detwiler Building.

---

EDITOR'S NOTE: As Dr. Harrower says, this is still somewhat of a "new" subject, and it is quite likely that the TEXAS MEDICAL JOURNAL can serve its readers to better advantage by offering them more direct personal information. So we have arranged to conduct a correspondence clinic for the benefit of our readers, in which cases of definite or fancied endocrinous disorder may be considered and suggestions made regarding their care. To avoid unnecessary inconvenience and delay it is requested that: (1) Only one case be mentioned in a letter; (2) the clinical data be as concise and *complete* as possible; (3) communications be written on one side of the paper, and that (4) two 2-cent stamps be enclosed—for forwarding the letter and the answer. Inquirers will hear direct from Dr. Harrower and several of these "clinics" will be printed following each installment of this series. It is believed that this feature alone will multiply the very obvious value that a series of this character, on so important and little appreciated a subject, can be to those who follow it from month to month.

Address all communications to "Clinic" in care of the TEXAS MEDICAL JOURNAL.

In June, "The Detection of the Minor Thyroid Dyscrasies." Don't miss it!

---

Experiments are being made by specialists at Johns Hopkins University Hospital which, if successful, will greatly aid in the resuscitation of persons apparently dead from drowning or asphyxiation. The new treatment is the injection of a serum to stimulate the blood to such an extent as will form a reaction on the heart.

